# Measles epidemiological surveillance system before and during the COVID-19 pandemic in Pernambuco, Brazil, 2018-2022: a descriptive evaluation

**DOI:** 10.1590/S2237-96222023000300008.EN

**Published:** 2023-11-27

**Authors:** Cinthia Regina Albuquerque de Souza, Lygia Carmen de Moraes Vanderlei, Paulo Germano de Frias

**Affiliations:** 1 Secretaria Estadual de Saúde de Pernambuco, Laboratório Central de Saúde Pública Dr. Milton Bezerra Sobral, Recife, PE, Brazil; 2 Instituto de Medicina Integral Prof. Fernando Figueira, Programa de Pós-Graduação em Avaliação em Saúde, Recife, PE, Brazil

**Keywords:** Health Evaluation, Epidemiological Monitoring, Measles, COVID-19, Communicable Diseases, Health Information System, Evaluación en Salud, Monitoreo Epidemiológico, Sarampión, COVID-19, Enfermedades Transmisibles, Sistema de Información en Salud, Avaliação em Saúde, Monitoramento Epidemiológico, Sarampo, Covid-19, Doenças Transmissíveis, Sistemas de Informação em Saúde

## Abstract

**Objective:**

To evaluate the measles epidemiological surveillance system, before and during the COVID-19 pandemic in Pernambuco, Brazil.

**Methods:**

This was a descriptive evaluation of the quality (duplicity; completeness; consistency), timeliness and usefulness attributed, classified as excellent ≥ 90.0%, regular ≥ 70.0% and < 90.0%, and poor (< 70.0%). Data from the Notifiable Health Conditions Information System and Laboratory Environment Management System were used, before (03/11/2018-03/10/2020) and during (03/11/2020-03/10/2022) the pandemic.

**Results:**

1,548 suspected measles cases were registered (1,469 before and 79 during the pandemic). In the two periods studied, there were 11 and 1 duplicate records, average completeness in filling out the variables was 99.2% and 95.7%, while average consistency was 96.7% and 97.5%, respectively. Timeliness (receipt of samples, 16.2% and 33.0%. Release of results, 1.3% and 1.3%) and usefulness (43.5% and 24.4%) were poor.

**Conclusion:**

Quality was classified as excellent in the periods studied, timeliness and usefulness were classified as poor, signaling non-compliance with the purpose of the system.

## Study contributions

**Main results:**

The quality of data from the measles epidemiological surveillance system in Pernambuco was excellent, while its timeliness and usefulness were poor during both periods.

**Implications for services:**

The limited timeliness and, therefore, the low usefulness of the measles epidemiological surveillance system must be discussed in the three government spheres of health service management, with the aim of training the professionals involved, as well as monitoring and evaluating the system.

**Perspectives:**

Systematic monitoring and evaluation generates evidence that supports health service managers and workers in the timely identification of gaps that compromise the full fulfillment of the objectives proposed.

## Resumen

**Objetivo:**

Evaluar el sistema de vigilancia epidemiológica del sarampión, antes y durante la pandemia de covid-19 en Pernambuco, Brasil.

**Métodos:**

Evaluación descriptiva de los atributos calidad (duplicidad; exhaustividad; consistencia), oportunidad y utilidad, clasificados en óptimo ≥ 90,0%, regular ≥ 70,0% y < 90,0%, y malo (< 70,0%). Se utilizaron datos del Sistema de Información de Enfermedades de Declaración Obligatoria y Sistema de Gestión Ambiental de Laboratorio, antes (11/03/2018-10/03/2020) y durante la pandemia (11/03/2020-10/03/2022).

**Resultados:**

Se registraron 1.548 casos sospechosos de sarampión (1.469 antes y 79 durante la pandemia). En dos períodos estudiados hubo duplicidad de 11 y 1 registros, completitud en llenado de variables - medias de 99,2% y 95,7% - y consistencia - medias de 96,7% y 97,5% -, respectivamente. La puntualidad - recepción de muestra, 16,2% y 33,0%. Publicación de resultados, 1,3% y 1,3% - y utilidad - 43,5% y 24,4% - fueron malas.

**Conclusión:**

La calidad fue calificada como óptima pero la oportunidad y la utilidad, como malas, indicando incumplimiento del propósito del sistema.

## Resumo

**Objetivo:**

Avaliar o sistema de vigilância epidemiológica do sarampo, antes e durante a pandemia de covid-19, Pernambuco, Brasil.

**Métodos:**

Avaliação descritiva dos atributos de qualidade (duplicidade; completude; consistência), oportunidade e utilidade, considerados ótimos quando ≥ 90,0%, regulares quando ≥ 70,0% e < 90,0%, e ruins quando < 70,0. Foram utilizados dados do Sistema de Informação de Agravos de Notificação e do Sistema Gerenciador de Ambiente Laboratorial, antes (11/3/2018-10/3/2020) e durante a pandemia (11/3/2020-10/3/2022).

**Resultados:**

1.548 casos registrados suspeitos de sarampo (1.469 antes; 79 durante a pandemia). Nos dois períodos estudados, houve duplicidade de 11 e 1 registros, completude no preenchimento das variáveis - médias, 99,2% e 95,7% - e consistência - médias, 96,7% e 97,5% -, respectivamente. A oportunidade - recebimento da amostra, 16,2% e 33,0%; liberação de resultados, 1,3% e 1,3% - e a utilidade - 43,5% e 24,4% - mostraram-se ruins.

**Conclusão:**

A qualidade do sistema classificou-se como ótima, mas sua oportunidade e utilidade, ruins, sinalizando descumprimento de sua finalidade.

## INTRODUCTION

Measles is a viral disease, with universal distribution, endemic in large urban conglomerates where vaccination coverage is not homogeneous.^1^ ^),(^ ^2^ Its prevention requires, in particular, 95% vaccination coverage, isolation of suspected/confirmed cases and selective immunization of contacts within 72 hours.^1^

In Brazil, measles has been a notifiable disease since 1968 and, with the creation of the national immunization program in 1975, the first national measles vaccination campaign in 1992, along with surveillance, cases and deaths decreased consistently until the certification of its elimination in 2016.^3^ ^),(^ ^4^ However, two years after that achievement, the virus began circulating again and 10,346 cases were confirmed in the Brazil, four of them in Pernambuco; 12 months later, transmission remained active and circulation was endemic.^5^

With the reintroduction of the virus, epidemiological surveillance sought to immediately identify and report every suspected case on the Notifiable Health Conditions Information System (*Sistema de Informação de Agravos de Notificação* - SINAN), and establish appropriate control measures.^5^ SINAN system data is routinely generated by the epidemiological surveillance system, providing essential information for analyzing the population’s health situation, developing public policies, establishing priorities and proposing actions to address infections, diseases and related health problems, including epidemics.^5^ ^),(^ ^6^

Given the relevance of measles and the effectiveness of measures to control it, studies have been carried out using data from the epidemiological surveillance system;^5^ ^),(^ ^7^ however, a complex national scenario, revealed cuts in public expenditure, especially in Primary Health Care, became even more so with the declaration of a public health emergency of international concern due to COVID-19.

This raised imperative issues for the surveillance system, given the concentration of efforts by central public health laboratories (*Laboratórios Centrais de Saúde Pública* - LACEN) to diagnose the pandemic virus. Furthermore, fake news spread by anti-vaccine groups has created further difficulties both for surveillance and for the Brazilian National Health System (*Sistema Único de Saúde* - SUS), making the population immunologically vulnerable^8^ ^),(^ ^9^ and posing new challenges for disease prevention and control, particularly vaccine-preventable diseases.

Evaluations of the surveillance system in the period before and during the pandemic are essential to understand the implications of COVID-19 for the most diverse health problems. These evaluations can focus on system coverage,^10^ case underreporting and/or data quality and, especially, on certifying the reliability of the information made available^11^ or even aspects of the completeness of the system.^12^ One of the most used models to evaluate the health information systems is that of the United States Centers for Disease Control and Prevention (CDC), the analysis criteria of which enable comparisons between different countries and health surveillance policies.^13^

The objective of this study was to evaluate the data quality, timeliness, and usefulness attributes of the measles epidemiological surveillance system, before and during the COVID-19 pandemic in the state of Pernambuco, Brazil.

## METHODS

*Design*

A descriptive evaluation of the measles epidemiological surveillance system was performed, considering the selected attributes: data quality, timeliness and usefulness, according to the document entitled Updated Guidelines for Evaluating Public Health Surveillance Systems, published by the CDC.^13^ The Guidelines discuss analysis of quantitative and qualitative characteristics relevant to a surveillance system, based on parameters and evaluation criteria for each attribute.^13^

*Context*

The evaluation in question related to the state of Pernambuco, located in Northeast Brazil, where the estimated population is 9,674,793 inhabitants, distributed over 184 municipalities (14 of them in the metropolitan region of the state capital Recife) covered by 12 Regional Health Departments (*Gerências Regionais de Saúde* - GERES),^14^ which, in turn, are comprised of 2,750 active reporting health facilities. Furthermore, Pernambuco has a LACEN, the Dr. Milton Bezerra Sobral Central Public Health Laboratory, subordinated to the Pernambuco State Health Department Executive Secretariat for Health Surveillance and Primary Care. Pernambuco’s LACEN, located in the center of the capital Recife, coordinates the state’s network of public and private laboratories. It is responsible for laboratory analyses and is organized according to the importance of diseases and health conditions. Measles diagnosis is only carried out at the LACEN, which is responsible for regulating collection, packaging, conservation and transport of samples. The procedures to be adopted until the samples are received by the GERES are the responsibility of the municipalities of origin, although some closer to the capital send them directly to the LACEN. Samples from the GERES, or municipalities, are received by the LACEN every day of the week, but only during the daytime.

*Participants and sample size*

The study involved all measles cases resident in the state of Pernambuco, reported and recorded on the systems used. Reported cases of occurrence in the state among residents of from other Brazilian states were excluded. The data analyzed referred to two defined periods: before the COVID-19 pandemic, from March 11, 2018 to March 10, 2020; and during the pandemic, from March 11, 2020 to March 10, 2022.

*Data source*

As a data source, we used (i) the SINAN, the system responsible for collecting, transmitting and disseminating data for epidemiological surveillance, and (ii) the Laboratory Environment Management System of the Pernambuco Laboratory Network (*Sistema Gerenciador de Ambiente Laboratorial da Rede Pernambucana de Laboratórios* - GAL/RPELAB), the latter being coordinated by the LACEN. The GAL system contains laboratory information and allows integration with surveillance systems, allowing interoperability with the SINAN.^15^ ^),(^ ^16^ Data from the SINAN and GAL databases were accessed by the researchers in April 2022, at the Pernambuco State Health Department.

*Definition of the attributes and variables used*

The “data quality” attribute involves the completeness and validity of the data recorded on a surveillance system. Our analysis verified (i) duplicity, defined as the degree of repetition of the same reported case within the universe of records, (ii) completeness, defined as the degree of completeness of the variable analyzed, and (iii) consistency, the degree to which related variables present coherent and non-contradictory values.^13^

The “timeliness” attribute corresponds to the speed of the process between the surveillance system stages, evaluated in terms of availability of information on the health-related event, for planning and/or carrying out immediate disease prevention-control and control-intervention actions.^13^ Timeliness was examined from two perspectives: timeliness of receiving the sample, which is the difference between the date of receipt of the sample and the date the case is reported, with samples received within one day being considered timely; and timeliness of release of the result, which is the difference between the date of release of the result and the date the case is reported, with results released within two days being considered timely.

The “usefulness” attribute means the ability to prevent and control measles and the implications of adverse events on public health, based on the criterion of fulfilling the objective of maintaining relevant control measures and monitoring the risk conditions of the disease, in the sense of eliminating it.^13^ Selective immunization within 72 hours of contact was evaluated.

The variables considered in the study are defined below, according to attributes of the measles epidemiological surveillance system:

a) Duplicity

- patient’s name;

- date of birth;

- mother’s name; and

- SUS Card number.

b) Completeness

- health center;

- date of symptom onset;

- patient’s name;

- date of birth;

- age;

- sex;

- race/skin color;

- municipality of residence;

- date of investigation;

- suspected case vaccination coverage;

- rash onset date; and

- fever onset date.

c) Consistency

- age *versus* schooling;

- case reporting date *versus* symptom onset date;

- suspected case vaccination coverage *versus* date of last dose; and

- case reporting date *versus* result release date.

d) Timeliness

- of the receipt of the sample (sample receipt date and case reporting date); and

- of result release (result release date and case reporting date).

e) Usefulness (selective immunization performed within 72 hours).

Box 1 shows the matrix of indicators and parameters for evaluating the measles epidemiological surveillance system according to its “data quality”, “timeliness” and “usefulness” attributes.

**Box 1:** Matrix of indicators and parameters for evaluating the measles epidemiological surveillance system according to its “data quality”, “timeliness” and “usefulness” attributes, Pernambuco, Brazil, 2018-2022

Attribute	Indicator	Calculation	Parameter
Data quality/duplicity	Proportion of duplicated records	Total cases with duplicated records/total reported cases x 100	Excellent: < 1.0% Good: 1.0% - 2.9% Regular: 3.0% - 5.0% Poor: > 5.0%
Data quality/completeness	Proportion of reported cases with field filled out (except *unknown* and left *blank*)	Number of reported cases with field filled out (except unknown and left blank)/total reported cases x 100	Excellent: ≥ 90.0% Regular: ≥ 70.0% and < 90.0% Poor: < 70.0%
Data quality/consistency	Proportion of reported cases with related variables filled out with coherent values	Number of reported cases with coherent related variables/total reported cases x 100	Excellent: ≥ 90.0% Regular: ≥ 70.0% and < 90.0% Poor: < 70.0%
Timeliness of receipt of sample	Proportion of cases with period of up to one day between case reporting and receipt of sample	Number of cases with sample received in up to one day after reporting/total reported cases x 100	Excellent: ≥ 90.0% Regular: ≥ 70.0% and < 90.0% Poor: < 70.0%
Timeliness of result release	Proportion of cases with period of up to two days between case reporting and result release	Number of cases with result release in up to 2 days following case reporting/total reported cases x 100	Excellent: ≥ 90.0% Regular: ≥ 70.0% and < 90.0% Poor: < 70.0%
Usefulness	Proportion of cases with selective immunization performed in up to 72 hours	Number of cases with selective immunization performed in up to 72h/total reported cases x 100	Excellent: ≥ 90.0% Regular: ≥ 70.0% and < 90.0% Poor: < 70.0%

*Statistical methods*

Absolute numbers and their proportions were used for each variable representing the attributes studied, in addition to the average completeness and consistency of the data.

We used the Microsoft® Office Excel 2011 pivot table for processing, data analysis and exclusion of duplicates, considering a duplicate record to be the same reported case or the same result, on the same day and for the same person. Once this checking was completed, we tabulated the aforementioned attributes.

*Ethical aspects*

The study project was submitted for analysis by the Instituto de Medicina Integral Prof. Fernando Figueira Ethics Committee for Research Involving Human Beings: Certificate of Submission for Ethical Appraisal (*Certificado de Apresentação para Apreciação Ética* - CAAE) No. 53821121000005201; Opinion No. 5.170.964, dated December 16, 2021.

## RESULTS

In all, 1,548 suspected measles case were recorded in Pernambuco during the study period, 1,469 of which were before the COVID-19 pandemic and 79 during the pandemic period. Eleven (0.7%) duplicated reported cases, referring to the pre-pandemic period, and one (0.1%) duplicated reported case that occurred during the pandemic were excluded from the analysis, resulting in 1,536 records. Thus, duplicity was considered to be excellent before and during the pandemic.

The completeness of the 12 variables analyzed in the pre-pandemic period was considered to be excellent, while during the COVID-19 pandemic, 11 of the variables had excellent completeness. The “suspected case vaccination coverage” variable, related to clinical aspects, was poor, as only 41 (52.6%) suspected cases were had complete information (Table 1). As for consistency, still referring to the “data quality” attribute, of the four relationships studied, three were classified as excellent before and during the pandemic. The relationship between “suspected case vaccine coverage and date of last dose” was consistent, 578 (84.0%) consistent records were found for the pre-pandemic period and 36 (88.0%) during the pandemic, therefore being classified as regular (Table 2). Considering the cases of duplication observed and the estimated completeness and consistency averages, during the two periods studied, the measles epidemiological surveillance system was classified as being of excellent quality in terms of the data recorded.

**Table 1:** Completeness of the measles epidemiological surveillance system variables before and during the COVID-19 pandemic, Pernambuco, Brazil, 2018-2022

Variables	Before the pandemic n (%)^a^	Classification	During the pandemic n (%)^b^	Classification
Health Center	1,458 (100.0)	Excellent	78 (100.0)	Excellent
Patient’s name	1,458 (100.0)	Excellent	78 (100.0)	Excellent
Data of birth	1,419 (97.0)	Excellent	78 (100.0)	Excellent
Age	1,419 (97.0)	Excellent	78 (100.0)	Excellent
Sex	1,458 (100.0)	Excellent	78 (100.0)	Excellent
Race/skin color	1,449 (99.4)	Excellent	77 (98.7)	Excellent
Municipality of residence	1,458 (100.0)	Excellent	78 (100.0)	Excellent
Date of symptom onset	1,458 (100.0)	Excellent	78 (100.0)	Excellent
Date of investigation	1,456 (99.9)	Excellent	78 (100.0)	Excellent
Vaccination coverage of suspected cases	1,448 (99.3)	Excellent	41 (52.6)	Poor
Rash onset date	1,443 (99.0)	Excellent	77 (98.7)	Excellent
Fever onset date	1,437 (98.6)	Excellent	77 (98.7)	Excellent
Average data completeness	1,447 (99.2)	Excellent	75 (98.7)	Excellent

a) Before the COVID-19 pandemic, from 11/3/2018 to 10/3/2020; b) During the COVID-19 pandemic, from 11/3/2020 to 10/3/2022.

**Table 2:** Consistence of the measles epidemiological surveillance system before and during the COVID-19 pandemic, Pernambuco, Brazil, 2018-2022

Related variables	Before the pandemic n (%)^a^	Classification	During the pandemic n (%)^b^	Classification
Age *versus* schooling	1,458 (100.0)	Excellent	78 (100.0)	Excellent
Case reporting date *versus* symptom onset date	1,458 (100.0)	Excellent	78 (100.0)	Excellent
Suspected case vaccination coverage^c^ *versus* date of last dose	578 (84.0)	Regular	36 (88.0)	Regular
Case reporting date *versus* result release date	363 (95.0)	Excellent	3 (100.0)	Excellent
Average data consistency	964 (96.7)	Excellent	49 (97.5)	Excellent

a) Before the COVID-19 pandemic, from 11/3/2018 to 10/3/2020; b) During the COVID-19 pandemic, from 11/3/2020 to 10/3/2022; c) Suspected case vaccination coverage before the pandemic, 689; during the pandemic, 41.

There were 383 confirmed cases of measles (229 in the interior region of Pernambuco and 154 in the metropolitan region of Recife) before the pandemic, and three (one in the interior region and two in the metropolitan region) during the pandemic. Only 62 (16.2%) samples (42 from the metropolitan region and 20 from the interior) were considered to be timely before the pandemic, and one sample from the metropolitan region (33.0%) during the pandemic. Only five (1.3%) samples had their results released within up to two days after the cases were reported, in the pre-pandemic period, with timeliness being considered poor in both periods (Table 3).

**Table 3:** Timeliness of the measles epidemiological surveillance system before and during the COVID-19, Pernambuco, Brazil, 2018-2022

Timeliness	Before the pandemic n (%)^a^	During the pandemic n (%)^b^	Total n (%)
Samples received	383 (100.0)	3 (100.0)	386 (100.0)
Up to one day after case reported^c^	62 (16.2)	1 (33.0)	63 (16.3)
More than one day after case reported	321 (83.8)	2 (67.0)	323 (83.7)
Results released	383 (100.0)	3 (100.0)	386 (100.0)
Up to two days after case reported	5 (1.3)	-	5 (1.3)
More than two days after case reported	378 (98.7)	3 (100.0)	381 (98.7)

a) Before the COVID-19 pandemic, from 11/3/2018 to 10/3/2020; b) During the COVID-19 pandemic, from 11/3/2020 to 10/3/2022; c) Before the pandemic, of the 62 samples received in up to one day after case reported, 42 came from the metropolitan region of Recife and 20 from the interior region of the state of Pernambuco; during the pandemic, the only sample received in up to one day after case reported came from the metropolitan region of Recife.

With regard to the “usefulness” attribute of the measles epidemiological surveillance system, selective immunization was carried out in 889 (57.9%) of the cases under investigation, throughout the study period. However, only 653 (42.5%) occurred within 72 hours, this being the ideal time for controlling transmissibility; 634 (43.5%) in the pre-pandemic period and 19 (24.4%) during the COVID-19 pandemic. As such this attribute was considered to be poor before and during the pandemic (Table 4).

**Table 4:** Usefulness of the measles epidemiological surveillance system before and after the COVID-19 pandemic, Pernambuco, Brazil, 2018-2022

Selective immunization	Before the pandemic n (%)^a^	During the pandemic n (%)^b^	Total n (%)
Performed	853 (58.5)	36 (46.2)	889 (57.9)
In up to 72 hours	634 (43.5)	19 (24.4)	653 (42.5)
After 72 hours	170 (11.7)	13 (16.7)	183 (11.9)
Unknown/left blank	49 (3.3)	4 (5.1)	53 (3.4)
Not performed	159 (10.9)	9 (11.5)	168 (10.9)
Unknown/left blank	340 (23.4)	20 (25.6)	360 (23.5)
Not performed, all contacts vaccinated	90 (6.2)	12 (15.4)	102 (6.6)
Not performed, no history of contact	16 (1.1)	1 (1.3)	17 (1.1)
Total	1,458 (100.0)	78 (100.0)	1,536 (100.0)

a) Before the COVID-19 pandemic, from 11/3/2018 to 10/3/2020; b) During the COVID-19 pandemic, from 11/3/2020 to 10/3/2022.

## DISCUSSION

The evaluation of the three attributes of the measles epidemiological surveillance system - quality, timeliness and usefulness -, in both periods of the study, showed excellent data quality, with regard to duplicity, completeness in filling out variables and consistency of records; however, timeliness of receiving the samples and releasing the results proved to be poor, as did usefulness in preventing and controlling measles, since selective immunization was not carried out within the recommended time frame. These findings signaled that, from the perspective of fulfilling the system’s purpose, the control and elimination of measles in Brazil have faced concrete difficulties in achieving their objectives.

Among the limitations of the study are those inherent to the use of secondary data from the information systems accessed: underreporting, for example, produces biases, by underestimating the number of people with measles and their contacts. Furthermore, underreporting could have intensified in the context of COVID-19, due to operational losses caused by the pandemic: the complexity of analyzing the measles epidemiological surveillance system at the LACEN, associated with the prioritization of COVID-19, could have further affected the true measurement of the indicators. Another limitation of the study refers to the unavailability of studies on the quality, timeliness and usefulness of measles data, making comparison with other research difficult.

In both periods of the study, the variables presented the same final classification, despite the significant quantitative difference in suspected and confirmed cases - with the exception of the completeness of the variable regarding the filling out of information on suspected cases who were given vaccine against measles, the only variable classified as excellent in the period before the pandemic and poor during the pandemic. This was probably influenced by the negative effects of the pandemic on epidemiological surveillance and the health system.^17^ ^),(^ ^18^ ^)^ Unlike the findings of our study, descriptive research carried out in João Pessoa, capital of the state of Paraíba, between 2001 and 2019, when addressing the completeness of reported cases of leprosy on the SINAN system, showed variations in filling out the variables, ranging from excellent to very poor, while consistency fluctuated from poor to excellent, thus making an adequate epidemiological analysis of the disease difficult.^11^

With the aim of minimizing problems arising from the low quality of SINAN data and keeping them at levels classified as excellent, analytical evaluation research into implementation of this system in Pernambuco, carried out in 2014, showed the relevance of monitoring its quality over short intervals.^6^ Data quality control routines, developed by Health Departments, certainly contribute to improved quality, as previously demonstrated.^6^ This quality control procedure, also found in research on another health condition, is possibly expressed in the results of the present study regarding duplicity.^11^

Ideally, epidemiological surveillance systems should support data acquisition, analysis and dissemination in a timely, flexible, measurable and scaleable manner - unlike the measles epidemiological surveillance system in Pernambuco, the timeliness of which was classified as poor. Many systems, such as the SINAN, depend on specific approaches for each health condition, inhibit efficiency and interoperability, insufficiently valuing user needs for data management, analysis, visualization and dissemination.^1^ ^),(^ ^19^ These problems become significantly worse during a pandemic, requiring greater adaptability for quick responses. In this situation, adequate infrastructures are required for epidemiological surveillance systems and health services, in order to strengthen them for data collection, processing and dissemination.^20^ ^),(^ ^21^

In this evaluation of the measles epidemiological surveillance system in Pernambuco, we found that a system with poor timeliness will have its usefulness impaired, in terms of responding to outbreaks in a timely and standardized manner,^22^ not achieving its objective of maintaining the elimination of measles through active, sensitive and appropriate surveillance.^1^ ^)^ In the case studied, centralization of laboratory diagnosis of measles at the LACEN, located in the state capital, may have contributed to poor timeliness in receiving samples, considering the operational difficulties related to transport and receipt of samples, which only takes place during the day.

Improving an epidemiological surveillance system is not a simple task in countries of continental size, as was the case in the United States in 2014, when, after 25 years of outdated standards, the national notifiable diseases surveillance system brought together state governments, the CDC and epidemiologists, with the aim of modernizing it. Data in that country were made compatible and standardized; electronic messaging systems were created to transfer data in a timely manner. Two years later, only ten U.S. states (25% of the country’s population) were using the new standards.^19^

The COVID-19 pandemic had repercussions on routine vaccination, with a considerable decrease in coverage of the first and second doses of the measles, mumps and rubella (MMR) vaccine, in Brazil and around the world.^8^ ^),(^ ^12^ ^),(^ ^23^ The negative effects of the pandemic emergency extended to the diagnosis of other diseases.^17^ ^),(^ ^18^ ^),(^ ^24^ ^)-(^ ^26^ In Brazil, meningitis, leprosy, tuberculosis, dengue fever and domestic violence even showed a decrease in compulsory reporting in 2020, due to the reduction in diagnoses or operational losses in surveillance programs, caused by the pandemic,^17^ ^),(^ ^18^ ^),(^ ^24^ ^)-(^ ^26^ ^)^ a fact which may possibly have been repeated in relation to reported suspected measles cases.

The high number of measles cases worldwide has the potential to trigger epidemics, which reaffirms the challenges of expanding vaccination coverage and enabling receptive and adequate epidemiological surveillance.^7^ ^),(^ ^12^ ^),(^ ^27^ ^)^ This reality is particularly relevant in Brazil, which had already been experiencing cases and hospitalizations due to measles associated with the massive presence of immigrants in the north of the country, associated with difficulty in accessing health services, low vaccination coverage and no vaccination card requirement, factors that have favored the spread of the disease.^7^ ^),(^ ^23^

We conclude that, despite the quality of the data analyzed being excellent, before and during the COVID-19 pandemic, the timeliness and usefulness of the surveillance system in question were poor, pointing to failure to fulfill its purpose. In view of these findings, it is recommended that discussions be held between the three governmental spheres of health service management, on structural aspects and work processes associated with the training of professionals involved, monitoring and evaluation activities, so as to be able to contribute to improving results and improving the measles epidemiological surveillance system in Pernambuco.
